# Rota–Baxter operators and post-Lie algebra structures on semisimple Lie algebras

**DOI:** 10.1080/00927872.2018.1536206

**Published:** 2019-01-11

**Authors:** Dietrich Burde, Vsevolod Gubarev

**Affiliations:** Fakultät für Mathematik, Universität Wien, Wien, Austria

**Keywords:** Post-Lie algebra, Rota–Baxter operator, 17B20, 17D25, 16T25

## Abstract

Rota–Baxter operators *R* of weight 1 on n are in bijective correspondence to post-Lie algebra structures on pairs (g,n), where n is complete. We use such Rota–Baxter operators to study the existence and classification of post-Lie algebra structures on pairs of Lie algebras (g,n), where n is semisimple. We show that for semisimple g and n, with g or n simple, the existence of a post-Lie algebra structure on such a pair (g,n) implies that g and n are isomorphic, and hence both simple. If n is semisimple, but g is not, it becomes much harder to classify post-Lie algebra structures on (g,n), or even to determine the Lie algebras g which can arise. Here only the case $n=sl_{2}(C)$ was studied. In this paper, we determine all Lie algebras g such that there exists a post-Lie algebra structure on (g,n) with $n=sl_{2}(C)⊕sl_{2}(C)$.

## 1. Introduction

Rota–Baxter operators were introduced by Baxter [3] in 1960 as a formal generalization of integration by parts for solving an analytic formula in probability theory. Such operators R:A→A are defined on an algebra *A* by the identity

R(x)R(y)=R(R(x)y+xR(y)+λxy)

for all x,y∈A, where λ is a scalar, called the *weight* of *R*. These operators were then further investigated, by Rota [30], Atkinson [1], Cartier [16] and others. In the 1980s, these operators were studied in integrable systems in the context of classical and modified Yang–Baxter equations [33,4]. Since the late 1990s, the study of Rota–Baxter operators has made great progress in many areas, both in theory and in applications [25,2,22,20,21,5,19].

Post-Lie algebras and post-Lie algebra structures also arise in many areas, e.g., in differential geometry and the study of geometric structures on Lie groups. Here, post-Lie algebras arise as a natural common generalization of pre-Lie algebras [23,26,32,6,7,8] and LR-algebras [9,10], in the context of nil-affine actions of Lie groups, see [11]. A detailed account of the differential geometric context of post-Lie algebras is also given in [18]. On the other hand, post-Lie algebras have been introduced by Vallette [34] in connection with the homology of partition posets and the study of Koszul operads. They have been studied by several authors in various contexts, e.g., for algebraic operad triples [28], in connection with modified Yang–Baxter equations, Rota–Baxter operators, universal enveloping algebras, double Lie algebras, *R*-matrices, isospectral flows, Lie-Butcher series and many other topics [2,18,19]. There are several results on the existence and classification of post-Lie algebra structures, in particular on commutative post-Lie algebra structures [13–15].

It is well-known [2] that Rota–Baxter operators *R* of weight 1 on n are in bijective correspondence to post-Lie algebra structures on pairs (g,n), where n is complete. In fact, RB-operators always yield PA-structures. So it is possible (and desirable) to use results on RB-operators for the existence and classification of post-Lie algebra structures.

The paper is organized as follows. In Section 2 we give basic definitions of RB-operators and PA-structures on pairs of Lie algebras. We summarize several useful results. For a complete Lie algebra n there is a bijection between PA-structures on (g,n) and RB-operators of weight 1 on n. The PA-structure is given by x·y={R(x),y}. Here we study the kernels of *R* and R+id. If g and n are not isomorphic, then both *R* and R+id have a nontrivial kernel. Moreover, if one of g or n is not solvable, then at least one of ker(R) and ker(R+id) is nontrivial.

In Section 3, we complete the classification of PA-structures on pairs of semisimple Lie algebras (g,n), where either g or n is simple. We already have shown the following in [11]. If g is simple, and there exists a PA-structure on (g,n), then also n is simple, and we have g≅n with x·y=0 or x·y=[x,y]. Here we deal now with the case that n is simple. Again it follows that g and n are isomorphic. The proof via RB-operators uses results of Koszul [27] and Onishchik [29]. We also show a result concerning semisimple decompositions of Lie algebras. Suppose that $g=s_{1}+s_{2}$ is the vector space sum of two semisimple subalgebras of g. Then g is semisimple. As a corollary we show that the existence of a PA-structure on (g,n) for g semisimple and n complete implies that n is semisimple.

In Section 4, we determine all Lie algebras g which can arise by PA-structures on (g,n) with $n=sl_{2}(C)⊕sl_{2}(C)$. This turns out to be much more complicated than the case $n=sl_{2}(C)$, which we have done in [11]. By Theorem 3.3 of [12], g cannot be solvable unimodular. On the other hand, the result we obtain shows that there are more restrictions than that.

## 2. Preliminaries

Let *A* be a nonassociative algebra over a field *K* in the sense of Schafer [31], with *K*-bilinear product A×A→A,(a,b)↦ab. We will assume that *K* is an arbitrary field of characteristic zero, if not said otherwise.

Definition 2.1. Let λ∈K. A linear operator R:A→A satisfying the identity

(1) R(x)R(y)=R(R(x)y+xR(y)+λxy)

for all x,y∈A is called a *Rota–Baxter operator on A of weight*λ, or just *RB-operator*.

Two obvious examples are given by *R* = 0 and R=λid, for an arbitrary nonassociative algebra. These are called the *trivial* RB-operators. The following elementary lemma was shown in [22], Proposition 1.1.12.

Lemma 2.2. *Let R be an RB-operator on A of weight λ. Then*−R−λid*is an RB-operator on A of weight λ, and*$\lambda^{-1}R$*is an RB-operator on A of weight 1 for all*λ≠0.

It is also easy to verify the following results.

Proposition 2.3. [5] *Let R be an RB-operator on A of weight λ and*ψ∈Aut(A)*. Then*$R^{\left(\psi \right)}=\psi^{-1}R\psi$*is an RB-operator on A of weight*λ.

Proposition 2.4. [22] *Let B be a countable direct sum of an algebra A. Then the operator R defined on B by*

$$R((a_{1},a_{2},\ldots ,a_{n},\ldots ))=(0,a_{1},a_{1}+a_{2},a_{1}+a_{2}+a_{3},\ldots )$$

*is an RB-operator on B of weight 1.*

Proposition 2.5. *Let*B=A⊕A*and*ψ∈Aut(A)*. Then the operator R defined on B by*

(2) $$R((a_{1},a_{2}))=(0,\psi (a_{1}))$$

*is an RB-operator on B of weight 1. Furthermore the operator R defined on B by*

(3) $$R((a_{1},a_{2}))=(-a_{1},-\psi (a_{1}))$$

*is an RB-operator on B of weight 1.*

Proof. Let $x=(a_{1},a_{2})$ and $y=(b_{1},b_{2})$. Then we have

$$\begin{array}{l} R(R(x)y+xR(y)+\lambda xy)=R((0,\psi (a_{1})b_{2}+(0,a_{2}\psi (b_{1}))+(a_{1}b_{1},a_{2}b_{2}))\\ =(0,\psi (a_{1}b_{1}))\\ =(0,\psi (a_{1})\psi (b_{1}))\\ =R(x)R(y). \end{array}$$

The second claim follows similarly.□

Proposition 2.6. [25] *Let*$A=A_{1}⊕A_{2}$*, R_1_ be an RB-operator of weight*λ*on A_1_, R_2_ be an RB-operator of weight*λ*on A_2_. Then the operator*R:A→A*defined by*$R((a_{1},a_{2}))=(R_{1}(a_{1}),R_{2}(a_{2}))$*is an RB-operator of weight*λ*on A.*

Proposition 2.7. [22] *Let*$A=A_{1}\dot{+}A_{2}$*be the direct vector space sum of two subalgebras. Then the operator R defined on A by*

(4) $$R(a_{1}+a_{2})=-\lambda a_{2}$$

*for*$a_{1}\in A_{1}$*and*$a_{2}\in A_{2}$*is an RB-operator on A of weight*λ.

We call such an operator *split*, with subalgebras *A*_1_ and *A*_2_. Note that the set of all split RB-operators on *A* is in bijective correspondence with all decompositions $A=A_{1}\dot{+}A_{2}$ as a direct sum of subalgebras.

Lemma 2.8. [5] *Let R be an RB-operator of nonzero weight*λ*on an algebra A. Then R is split if and only if*R(R+λid)=0.

Lemma 2.9. *Let*$A=A_{-}\dot{+}A_{0}\dot{+}A_{+}$*be a direct vector space sum of subalgebras of A. Suppose that R is an RB-operator of weight*λ*on A_0_,*$A_{-}$*is an*$\left(R+\text{id})(A_{0}\right)$*-module and*$A_{+}$*is an*$R(A_{0})$*-module. Define an operator P on A by*

(5) $$P_{|A_{-}}=0,P_{|A_{0}}=R,P_{|A_{+}}=-\lambda \text{id}.$$

*Then P is an RB-operator on A of weight*λ.

Definition 2.10. Let *P* be an RB-operator on *A* defined as above such that not both $A_{-}$ and $A_{+}$ are zero. Then *P* is called *triangular-split*.

We also recall the definition of post-Lie algebra structures on a pair of Lie algebras (g,n) over *K*, see [11].

Definition 2.11. Let g=(V,[ ,]) and n=(V,{ ,}) be two Lie brackets on a vector space *V* over *K*. A *post-Lie algebra structure*, or *PA-structure* on the pair (g,n), is a *K*-bilinear product x·y satisfying the identities:

(6) x·y−y·x=[x,y]−{x,y},

(7) [x,y]·z=x·(y·z)−y·(x·z),

(8) x·{y,z}={x·y,z}+{y,x·z}

for all x,y,z∈V.

Define by L(x)(y)=x·y the left multiplication operator of the algebra A=(V,·). By (8), all *L*(*x*) are derivations of the Lie algebra (V,{,}). Moreover, by (7), the left multiplication

L:g→Der(n)⊆End(V),x↦L(x)

is a linear representation of g.

If n is abelian, then a post-Lie algebra structure on (g,n) corresponds to a *pre-Lie algebra structure* on g. In other words, if {x,y}=0 for all x,y∈V, then the conditions reduce to

$$\begin{array}{l} x\cdot y-y\cdot x=[x,y],\\ {}[x,y]\cdot z=x\cdot (y\cdot z)-y\cdot (x\cdot z), \end{array}$$

i.e., x·y is a *pre-Lie algebra structure* on the Lie algebra g, see [11].

Definition 2.12. Let x·y be a PA-structure on (g,n). If there exists a φ∈End(V) such that

x·y={φ(x),y}

for all x,y∈V, then x·y is called an *inner* PA-structure on (g,n).

The following result is proved in [2], Corollary 5.6.

Proposition 2.13. *Let*(n,{,},R)*be a Lie algebra together with a Rota–Baxter operator R of weight 1, i.e., a linear operator satisfying*

{R(x),R(y)}=R({R(x),y}+{x,R(y)}+{x,y})

*for all*x,y∈V*. Then*

x·y={R(x),y}

*defines an inner PA-structure on*(g,n)*, where the Lie bracket of*g*is given by*

(9) [x,y]={R(x),y}−{R(y),x}+{x,y}.

Note that ker(R) is a subalgebra of n. For x,y∈ker(R) we have R({x,y})=0. Recall that a Lie algebra is called *complete*, if it has trivial center and only inner derivations.

Proposition 2.14. *Let*n*be a Lie algebra with trivial center. Then any inner PA-structure on*(g,n)*arises by a Rota–Baxter operator of weight 1. Furthermore, if*n*is complete, then every PA-structure on*(g,n)*is inner.*

Proof. The first claim follows from Proposition 2.10 in [11]. By Lemma 2.9 in [11] every PA-structure on (g,n) with complete Lie algebra n is inner. The result can also be derived from the proof of Theorem 5.10 in [2].□

Corollary 2.15. *Let*n*be a complete Lie algebra. Then there is bijection between PA-structures on*(g,n)*and RB-operators of weight 1 on*n.

As we have seen, any inner PA-structure on (g,n) with Z(n)=0 arises by a Rota–Baxter operator of weight 1. For Lie algebra n with nontrivial center this need not be true.

Example 2.16. *Let*$\left(e_{1},e_{2},e_{3}\right)$*be a basis of V and*$n=r_{2}(K)⊕K$*with*$\{e_{1},e_{2}\}=e_{2}$*. Then*

$$\varphi =\left(\begin{array}{ccc} 1 & 0 & 0\\ 0 & -1 & 0\\ \alpha & \beta & \gamma \end{array}\right)$$

*defines an inner PA-structure on*(g,n)*by*x·y={φ(x),y}*with*g=n*, i.e., with*$[e_{1},e_{2}]=e_{2}$*. But*φ*is not always a Rota–Baxter operator of weight 1 for*n*. It is easy to see that this is the case if and only if*β=0.

Proposition 2.17. *Let*x·y*be an inner PA-structure arising from an RB-operator R on*n*of weight 1. Then R is also an RB-operator of weight 1 on*g*, i.e., it satisfies*

[R(x),R[y)]=R([R(x),y]+[x,R(y)]+[x,y])

*for all*x,y∈V.

Proof. Because of R([x,y])={R(x),R(y)} and the definition of [x,y] we have

$$\begin{array}{l} R([R(x),y]+[x,R(y)]+[x,y])=\{R(R(x)),R(y)\}+\{R(x),R(R(y))\}+\{R(x),R(y)\}\\ =[R(x),R(y)] \end{array}$$

for all x,y∈V.□

Corollary 2.18. *Let*x·y={R(x),y}*be a PA-structure on*(g,n)*defined by an RB-operator R of weight 1 on*n*. Denote by*$g_{i}$*be the Lie algebra structure on V defined by*

$$\begin{array}{l} {\left[x,y\right]}_{0}=\{x,y\},\\ {\left[x,y\right]}_{i+1}={\left[R(x),y\right]}_{i}-{\left[R(y),x\right]}_{i}+{\left[x,y\right]}_{i}, \end{array}$$

*for all*i≥0*. Then R defines a PA-structure on each pair*$\left(g_{i+1},g_{i}\right)$.

We have ${\left[x,y\right]}_{1}=[x,y]$, and both *R* and R+id are Lie algebra homomorphisms from $g_{i+1}$ to $g_{i}$, see Proposition 7 in [33]. Hence, we obtain a composition of homomorphisms

$$g_{i}\overset{R}{\underset{R+\text{id}}{\to }}g_{i-1}\overset{R}{\underset{R+\text{id}}{\to }}\cdots \overset{R}{\underset{R+\text{id}}{\to }}g_{0}$$

So the kernels $\ker(R^{i})$ and $\ker({\left(R+\text{id}\right)}^{i})$ are ideals in $g_{j}$ for all 1≤i≤j.

For a Lie algebra g, denote by $g^{\left(i\right)}$ the derived ideals defined by $g^{\left(1\right)}=g$ and $g^{\left(i+1\right)}=[g^{\left(i\right)},g^{\left(i\right)}]$ for i≥1. An immediate consequence of Proposition 2.13 is the following observation.

Proposition 2.19. *Let*x·y={R(x),y}*be a PA-structure on*(g,n)*defined by an RB-operator R of weight 1 on*n*. Then we have*$\dim g^{\left(i\right)}\leq \dim n^{\left(i\right)}$*for all*i≥1.

Corollary 2.20. *Let*x·y*be a PA-structure on*(g,n)*, where*n*is complete. Then we have*$\dim g^{\left(i\right)}\leq \dim n^{\left(i\right)}$*for all*i≥1*. In particular, if*n*is solvable, so is*g*, and if*g*is perfect, so is*n.

Proof. By Corollary 2.15 this follows from the proposition.□

Proposition 2.21. *Let*x·y={R(x),y}*be a PA-structure on*(g,n)*defined by an RB-operator R of weight 1 on*n*. Then the following holds:*

1. *If*g*and*n*are not isomorphic, then both R and*R+id*have a nontrivial kernel.*
2. *If either*g*or*n*is not solvable, then at least one of the operators R and*R+id*has a nontrivial kernel.*

Proof. For (1), assume that ker(R)=0. Then R:g→n is invertible, hence an isomorphism. This is a contradiction. The same is true for R+id. For (2) assume that ker(R)=ker(R+id)=0. Then *R* and R+id are isomorphisms from g to n, and g≅n. Then we can apply a result of Jacobson [24] to the automorphism $\psi :=(R+\text{id}){}^{\circ}R^{-1}$ of n, because n is not solvable. We obtain a nonzero fixed point x∈n, so that

$$0=\psi (x)-x=(R+\text{id})R^{-1}(x)-x=R^{-1}(x).$$

Since *R* is bijective, *x* = 0, a contradiction.□

Corollary 2.22. *Let*n*be a simple Lie algebra and R be an invertible RB-operator of nonzero weight*λ*on*n*. Then we have*R=−λid.

Proof. By rescaling we may assume that *R* has weight 1. We obtain a PA-structure on (g,n) by Proposition 2.13, with Lie bracket (9) on g. Since n is not solvable, either *R* or R+id have a nontrivial kernel. But ker(R)=0 by assumption, so that ker(R+id) is a nontrivial ideal of n. Hence we have R+id=0.□

## 3. PA-structures on pairs of semisimple Lie algebras

We will assume that all algebras in this section are finite-dimensional. Let x·y be a PA-structure on (g,n) over C, where g is simple and n is semisimple. Then n is also simple, and both g and n are isomorphic, see Proposition 4.9 in [11]. We have a similar result for n simple and g semisimple. However, its proof is more difficult than the first one.

Theorem 3.1. *Let*x·y*be a PA-structure on*(g,n)*over*C*, where*n*is simple and*g*is semisimple. Then*g*is also simple, and both*g*and*n*are isomorphic.*

Proof. By Corollary 2.15 we have x·y={R(x),y} for an RB-operator *R* of weight 1 on n. Assume that g and n are not isomorphic. By Proposition 2.21 (2) both ker(R) and ker(R+id) are proper nonzero ideals of g, with ker(R)∩ker(R+id)=0. So we have

g=ker(R)⊕ker(R+id)⊕s

with a semisimple ideal s. We have n=im(R)+im(R+id) because of x=R(−x)+(R+id)(x) for all x∈n, and

$$\begin{array}{l} \text{im}(R)≅g/\ker(R)≅\ker(R+\text{id})⊕s,\\ \text{im}(R+\text{id})≅g/\ker(R+\text{id})≅\ker(R)⊕s. \end{array}$$

This yields a semisimple decomposition

n=(ker(R+id)⊕s)+(ker(R)⊕s).

Suppose that s is nonzero. Then both summands are not simple. This is a contradiction to Theorem 4.2 in Onishchik’s paper [29], which says that at least one summand in a semisimple decomposition of a simple Lie algebra must be simple. Hence we obtain s=0,im(R)=ker(R+id),im(R+id)=im(R) and

$$n=\text{im}(R)\dot{+}\text{im}(R+\text{id}).$$

Then the main result of Koszul’s note [27] implies that n=im(R)⊕im(R+id), which is a contradiction to the simplicity of n. Hence g and n are isomorphic.□

If g is semisimple with only two simple summands, we can prove the same result for any field *K* of characteristic zero.

Proposition 3.2. *Let*x·y*be a PA-structure on*(g,n)*, where*n*is semisimple, and*$g=s_{1}⊕s_{2}$*is the direct sum of two simple ideals of*g*. Then*g*and*n*are isomorphic.*

The proof is the same as before. The only argument where we needed the complex numbers was the result of [29], which we do not need here.

Let $n=s_{1}⊕s_{2}$ be a direct sum of two simple isomorphic ideals $s_{1}$ and $s_{2}$. We would like to find all RB-operators of weight 1 on n such that g with bracket (9) is isomorphic to n.

Proposition 3.3. *All PA-structures on*(g,n)*with*$g≅n=s_{1}⊕s_{2}$*, where*$s_{1}$*and*$s_{2}$*simple isomorphic ideals of*n*, arise by the trivial RB-operators or by one of the following RB-operators R on*n*, and*ψ∈Aut(n),

$$\begin{array}{l} R((s_{1},s_{2}))=(-s_{1},-\psi (s_{1})),\\ R((s_{1},s_{2}))=(0,\psi (s_{1})),\\ R((s_{1},s_{2}))=(-s_{1},0)), \end{array}$$

*up to permuting the factors and application of*φ(R)=−R−id*to these operators.*

Proof. By Proposition 2.5 and Proposition 2.7 the given operators are RB-operators of weight 1 on n, because *R* is. By Proposition 2.21 at least one of ker(R) and ker(R+id) is nonzero. Suppose first that both ker(R) and ker(R+id) are zero. Then we have g=ker(R)⊕ker(R+id) and $n=\ker(R)\dot{+}\ker(R+\text{id})$. It is easy to see that ker(R) coincides with $s_{1}$ or $s_{2}$ by using the Theorem of Koszul [27]. Applying φ if necessary, we can assume that $\ker(R)=s_{2}$. Then again by Koszul’s result we have $R((s_{1},s_{2}))=(\psi_{1}(s_{1}),\psi_{2}(s_{1}))$ or $R((s_{1},s_{2}))=(\psi_{1}(s_{1}),0))$ for some $\psi_{1},\psi_{2}\in \text{Aut}(n)$. Since im(R)=ker(R+id) we either have $R((s_{1},s_{2}))=(-s_{1},-\psi (s_{1}))$ or $R((s_{1},s_{2}))=(-s_{1},0)$.

In the second case, one of the kernels is zero. Applying φ if necessary, we may assume that ker(R+id)=0 and $\ker(R)=s_{1}$. Then g/ker(R) is a simple Lie algebra, and −R−id is an invertible RB-operator of weight 1 on g/ker(R). By Corollary 2.22 we obtain −R−id=−id, hence *R* = 0 on g/ker(R). This implies $R^{2}=0$ on g. The projections of im(R) to $s_{1}$ and $s_{2}$ are either zero or an isomorphism on one factor. So we have R((s,0))=(0,ψ(s)) or $R((s,0))=(\psi_{1}(s),\psi_{2}(s))$ for some automorphisms $\psi ,\psi_{1},\psi_{2}$. But the second operator does not satisfy $R^{2}=0$, and hence is impossible. Therefore we are done.□

Proposition 3.4. *Let*x·y={R(x),y}*be a PA-structure on*(g,n)*defined by an RB-operator R of weight 1 on*n*. Let*$n_{1}=\ker(R^{n}),n_{2}=\ker{\left(R+\text{id}\right)}^{n},n_{3}=\text{im}(R^{n})\cap \text{im}({\left(R+\text{id}\right)}^{n})$*for*n=dim(V)*. Then*$n=n_{1}\dot{+}n_{2}\dot{+}n_{3}$*with*$\{n_{1},n_{3}\}\subseteq n_{1},\{n_{2},n_{3}\}\subseteq n_{2}$*, and*$n_{3}$*is solvable.*

Proof. We first show by induction that $\ker(R^{i})$ is a subalgebra of n, and that

$$\left\{\ker(R^{i}),\text{im}({\left(R+\text{id}\right)}^{i})\}\subseteq \ker(R^{i}\right)$$

for all i≥1. The case *i* = 1 goes as follows. We already know that ker(R) is a subalgebra of n. So we have to show that {ker(R),im(R+id)}⊆ker(R). Let x∈ker(R) and y∈n. Then by (6) we have

$$\begin{array}{l} \left\{x,(R+\text{id})(y)\}=\{x,R(y)\}+\{x,y\right\}\\ =[x,y]+\{y,R(x)\}\\ =[x,y], \end{array}$$

which is in ker(R), since this is an ideal in g. For the induction step i↦i+1 consider the iteration of the Lie bracket (9) for all i≥0, given by

$${\left[x,y\right]}_{i}={\left[x,y\right]}_{i+1}-{\left[R(x),y\right]}_{i}-{\left[x,R(y)\right]}_{i}$$

for all i≥0. Then

$$\begin{array}{l} \{x,y\}={\left[x,y\right]}_{1}-{\left[R(x),y\right]}_{0}-{\left[x,R(y)\right]}_{0}\\ ={\left[x,y\right]}_{2}-{\left[R^{2}(x),y\right]}_{0}-2{\left[R(x),y\right]}_{0}-2{\left[R(x),R(y)\right]}_{0}-2{\left[x,R(y)\right]}_{0}-{\left[x,R^{2}(y)\right]}_{0} \end{array}$$

and so on. Define a degree of a term ${\left[R^{l}(x),R^{k}(y)\right]}_{m}$ by *l* +* k* + *m*, and let $x,y\in \ker(R^{i+1})$. We can iterate the brackets, until the degree of every summand on the right-hand side will be greater than 3*i*, so that all summands either have a term $R^{l}(x)$ with *l* >* i*, or a term $R^{k}(y)$ with *k* >* i*, or all summands lie in ${\left[\ker(R^{i+1}),\ker(R^{i+1})\right]}_{i+1}$. By induction hypothesis, such terms will vanish for *l* >* i* or *k* >* i*, and since $\ker(R^{i+1})$ is an ideal in $g_{i+1}$, we have $\left\{x,y\}\in \ker(R^{i+1}\right)$, so that $\ker(R^{i+1})$ is a subalgebra of n. The induction step for the second claim follows similarly.

Since the image of a subalgebra under the action of an RB-operator is a subalgebra, $n_{1},n_{2}$ and their intersection $n_{3}$ are subalgebras of n. We want to show that $n=n_{1}\dot{+}n_{2}\dot{+}n_{3}$. Because of $\ker(R^{n})\cap \text{im}(R^{n})=0$ we have $n=\ker(R^{n})\dot{+}\text{im}(R^{n})$. In the same way we have $n=\ker({\left(R+\text{id}\right)}^{n})\dot{+}\text{im}({\left(R+\text{id}\right)}^{n})$. We obtain

$$\text{im}(R^{n})\cap \ker({\left(R+\text{id}\right)}^{n})\dot{+}\text{im}(R^{n})\cap \text{im}({\left(R+\text{id}\right)}^{n})\subseteq \text{im}(R^{n}).$$

We claim that $\ker({\left(R+\text{id}\right)}^{n})\subseteq \text{im}(R^{n})$, so that we have equality above. Indeed, for $x\in \ker({\left(R+\text{id}\right)}^{n})$ we have by the binomial formula

$$x+\left(\begin{array}{c} n\\ n-1 \end{array}\right)R(x)+\cdots +\left(\begin{array}{c} n\\ 1 \end{array}\right)R^{n-1}(x)=-R^{n}(x)\in \text{im}(R^{n}).$$

Applying $R^{n-1}$ we obtain $R^{n-1}(x)\in \text{im}(R^{n})$ and

$$x+nR(x)+\cdots +\left(\begin{array}{c} n\\ 2 \end{array}\right)R^{n-2}(x)\in \text{im}(R^{n}).$$

Iterating this we obtain $x\in \text{im}(R^{n})$. This yields

$$\begin{array}{l} n=\ker(R^{n})\dot{+}\text{im}(R^{n})\\ =\ker(R^{n})\dot{+}\ker({\left(R+\text{id}\right)}^{n})\dot{+}\text{im}(R^{n})\cap \text{im}({\left(R+\text{id}\right)}^{n})\\ =n_{1}\dot{+}n_{2}\dot{+}n_{3}. \end{array}$$

On $n_{3}$ both operators *R* and R+id are invertible. By Proposition 2.21 part (2) it follows that $n_{3}$ is solvable.□

Corollary 3.5. *The decomposition*$n=n_{1}\dot{+}n_{2}\dot{+}n_{3}$*induces a decomposition*$g_{i}=n_{1}\dot{+}n_{2}\dot{+}n_{3}$*for each*i≥1*with the same properties as in the Proposition. The Lie algebras*$\left(n_{j},{\left[,\right]}_{i}\right)$*and*$\left(n_{j},{\left[,\right]}_{0}\right)$*are isomorphic for j = 1, 2, 3.*

Proof. Since *R* and R+id are RB-operators on all $g_{i}$, we obtain the same decomposition with the same subalgebras. Note that R+id is invertible on $n_{1}$, *R* is invertible on $n_{2}$ and both are invertible on $n_{3}$. In order to show that $(n_{1},{\left[,\right]}_{i}$ is isomorphic to $(n_{1},{\left[,\right]}_{0}$, we consider a chain of isomorphisms

$$(n_{1},{\left[,\right]}_{n})\overset{R+\text{id}}{\to }(n_{1},{\left[,\right]}_{n-1})\overset{R+\text{id}}{\to }\cdots \overset{R+\text{id}}{\to }(n_{1},{\left[,\right]}_{0}).$$

In a similar way we can deal with $n_{2}$ and $n_{3}$.□

Proposition 3.6. *Let*$g=s_{1}+s_{2}$*be the vector space sum of two complex semisimple subalgebras of*g*. Then*g*is semisimple.*

Proof. Suppose that the claim is not true and let g be a counterexample of minimal dimension. Then g contains a nonzero abelian ideal a. Then we obtain

$$g/a=s_{1}/(s_{1}\cap a)+s_{2}/(s_{2}\cap a).$$

Since $s_{1}\cap a$ is an abelian ideal $s_{1}$, it must be zero, i.e., $s_{1}\cap a=0$. In the same way we have $s_{2}\cap a=0$. Hence we obtain a semisimple decomposition of g/a with dim(g/a)<dim(g). If g/a is semisimple, this is a contradiction to the minimality of the counterexample g. Otherwise we may assume that g has one-dimensional solvable radical. Then g is reductive, and by Theorem 3.2 of [29], there are no semisimple decompositions of a complex reductive non-semisimple Lie algebra. Hence we are done.□

Proposition 3.7. *Let*x·y={R(x),y}*be a PA-structure on*(g,n)*over*C*, where*n*is simple, defined by an RB-operator R of weight 1 on*n*, with associated Lie algebras*$g_{i}$*for*i=1,…,n=dim(V)*. Assume that*$g_{0}=n$*and*$g_{n}$*are semisimple. Then all*$g_{i}$*are isomorphic to*n.

Proof. Since $n_{1}$ and $n_{2}$ are kernels of homomorphisms, they are ideals in $g_{n}$. The quotient $g_{n}/(n_{1}+n_{2})≅n_{3}$ is semisimple and solvable by Proposition 3.4. Hence $n_{3}=0$, and we obtain $g_{n}=\ker(R^{n})⊕\ker({\left(R+\text{id}\right)}^{n})$. Because of Corollary 3.5 we have the decomposition $g_{i}=\ker(R^{n})\dot{+}\ker({\left(R+\text{id}\right)}^{n})$ for all *i* <* n*, where all Lie algebras $\left(\ker(R^{n}),{\left[,\right]}_{i}\right)$ are isomorphic, and all Lie algebras $\left(\ker({\left(R+\text{id}\right)}^{n}),{\left[,\right]}_{i}\right)$ are isomorphic. By Proposition 3.6 all $g_{i}$ are semisimple. By Koszul’s result [27], all $g_{i}$ are isomorphic.□

Proposition 3.8. *Suppose that there is a post-Lie algebra structure on*(g,n)*over*C*, where*g*is semisimple and*n*is complete. Then*n*must be semisimple.*

Proof. By Corollary 2.15 the PA-structure is given by x·y={R(x),y}, where *R* is an RB-operator of weight 1 on n. If at least one of ker(R) and ker(R+id) is trivial, we obtain g≅n by Proposition 2.21, part (1). Otherwise n=im(R)+im(R+id) is the sum of two nonzero semisimple subalgebras. By Proposition 3.6n is semisimple.□

## PA-structures on (g,n) with $n=sl_{2}(C)\times sl_{2}(C)$

4.

In [11], Proposition 4.7 we have shown that PA-structures with $n=sl_{2}(C)$ exist on (g,n) if and only if g is isomorphic to $sl_{2}(C)$, or to one of the solvable non-unimodular Lie algebras $r_{3,\lambda }(C)$ for λ∈C∖{−1}. In this section we want to show an analogous result for $n=sl_{2}(C)\times sl_{2}(C)$. Here we will use RB-operators on n and an explicit classification by Douglas and Repka [17] of all subalgebras of n. This classification is up to inner automorphisms, but we will only need the subalgebras up to isomorphisms. Let us fix a basis $\left(X_{1},Y_{1},H_{1},X_{2},Y_{2},H_{2}\right)$ of n consisting of the following 4 × 4 matrices:

$$X_{1}=E_{12},\;Y_{1}=E_{21},\;H_{1}=E_{11}-E_{22},\;X_{2}=E_{34},\;Y_{2}=E_{43},\;H_{2}=E_{33}-E_{44}.$$

We use in Table 1.

**Table 1.** Complex 3-dimensional Lie algebras.

g	Lie brackets
$C^{3}$	–
$n_{3}(C)$	$[e_{1},e_{2}]=e_{3}$
$r_{2}(C)⊕C$	$[e_{1},e_{2}]=e_{2}$
$r_{3}(C)$	$[e_{1},e_{2}]=e_{2},\;[e_{1},e_{3}]=e_{2}+e_{3}$
$r_{3,\lambda }(C),\;\lambda \neq 0$	$[e_{1},e_{2}]=e_{2},\;[e_{1},e_{3}]=\lambda e_{3}$
$sl_{2}(C)$	$[e_{1},e_{2}]=e_{3},\;[e_{1},e_{3}]=-2e_{1},\;[e_{2},e_{3}]=2e_{2}$

**Table 2.** Solvable subalgebras.

dim(h)	Representative	Isomorphism type
1	$〈X_{1}〉,\;〈H_{1}〉,\;〈X_{1}+X_{2}〉,\;〈X_{1}+H_{2}〉,\;〈H_{1}+aH_{2}〉,\;a\in C^{*}$	C
2	$〈X_{1},X_{2}〉,\;〈X_{1},H_{2}〉,\;〈H_{1},H_{2}〉$	$C^{2}$
2	$〈X_{1}+X_{2},H_{1}+H_{2}〉,\;〈X_{1},H_{1}+X_{2}〉,\;〈X_{1},H_{1}+aH_{2}〉,\;a\in C$	$r_{2}(C)$
3	$〈X_{1},X_{2},H_{1}+\lambda H_{2}〉,\;\lambda \neq 0$	$r_{3,\lambda }(C),\lambda \neq 0$
3	$〈X_{1},H_{1},H_{2}〉,\;〈X_{1},H_{1},X_{2}〉$	$r_{2}(C)⊕C$
4	$〈X_{1},H_{1},X_{2},H_{2}〉$	$r_{2}(C)⊕r_{2}(C)$

Among the family $r_{3,\lambda }(C),\lambda \neq 0$ there are still isomorphisms. In fact, $r_{3,\lambda }(C)≅r_{3,\mu }(C)$ if and only if $\mu =\lambda^{-1}$ or μ=λ. The list of subalgebras h of n is given as follows. We first list the solvable subalgebras, then the semisimple ones and the subalgebras with a nontrivial Levi decomposition.

Theorem 4.1. *Suppose that there exists a post-Lie algebra structure on*(g,n)*, where*$n=sl_{2}(C)⊕sl_{2}(C)$*. Then*g*is isomorphic to one of the following Lie algebras, and all these possibilities do occur*:

1. $sl_{2}(C)⊕sl_{2}(C)$.
2. $sl_{2}(C)⊕r_{3,\lambda }(C),\;\lambda \neq -1$.
3. $r_{3,\lambda }(C)⊕r_{3,\mu }(C),\;(\lambda ,\mu )\neq (-1,-1)$.
4. $r_{2}(C)⊕r_{2}(C)⊕r_{2}(C)$.
5. $r_{2}(C)⊕(C^{3}⋉C)=〈x_{1},\ldots ,x_{6}〉$*and Lie brackets, for*α≠0,β≠0,−1
  $$[x_{1},x_{2}]=x_{1},\;[x_{3},x_{6}]=x_{3},\;[x_{4},x_{6}]=\alpha x_{4},\;[x_{5},x_{6}]=\beta x_{5}.$$
6. $C⊕((r_{3,\lambda }(C)⊕C)⋉C)=〈x_{1},\ldots ,x_{6}〉$*and Lie brackets, for*λ≠0,α≠0,−1,
  $$[x_{2},x_{4}]=x_{2},\;[x_{3},x_{4}]=\lambda x_{3},\;[x_{3},x_{6}]=x_{3},\;[x_{5},x_{6}]=\alpha x_{5}.$$
7. $(r_{3,\lambda }(C)⊕C^{2})⋉C=〈x_{1},\ldots ,x_{6}〉$*and Lie brackets, for*$\lambda \neq 0,\alpha_{1},\alpha_{2}\neq 0$*, and*$\left(\lambda ,\alpha_{1},\alpha_{2})\neq (-1,\alpha_{1},-\alpha_{1}-1\right)$,
  $$[x_{1},x_{3}]=x_{1},\;[x_{2},x_{3}]=\lambda x_{2},\;[x_{2},x_{6}]=\alpha_{1}x_{2},\;[x_{4},x_{6}]=x_{4},\;[x_{5},x_{6}]=\alpha_{2}x_{5}.$$
8. $(C^{2}⊕C^{2})⋉C^{2}=〈x_{1},\ldots ,x_{6}〉$*and Lie brackets*
  $$\begin{array}{c} {}[x_{1},x_{5}]=x_{1},[x_{2},x_{5}]=\alpha_{2}x_{2},\;[x_{3},x_{5}]=\alpha_{4}x_{3},\;[x_{4},x_{5}]=\alpha_{6}x_{4},\\ {}[x_{1},x_{6}]=\alpha_{1}x_{1},\;[x_{2},x_{6}]=\alpha_{3}x_{2},\;[x_{3},x_{6}]=\alpha_{5}x_{3},\;[x_{4},x_{6}]=\alpha_{7}x_{4}, \end{array}$$

with one of the following conditions:

- $(a)\;\alpha_{3}=1,\;\alpha_{5}=\alpha_{1}\alpha_{7},\;\alpha_{6}=\alpha_{2}\alpha_{4},\alpha_{1}\alpha_{2}\neq 1,\;\alpha_{4},\alpha_{7}\neq 0,-1,$
- $(b)\;\alpha_{4}=\alpha_{1}-1,\;\alpha_{5}=-\alpha_{1},\;\alpha_{6}=\alpha_{2}(\alpha_{1}-1),\;\alpha_{7}=\alpha_{1}\alpha_{3}-\alpha_{1}^{2}\alpha_{2}-\alpha_{3},\;\alpha_{3}-\alpha_{1}\alpha_{2}\neq 0,\;\alpha_{1}\neq 0,1.$

Proof. By Corollary 2.15 it is enough to consider the RB-operators *R* of weight 1 on n. Then ker(R) and ker(R+id) are ideals in g. If *R* is trivial, or one of the kernels is trivial, then we have g≅n, which is type (1). So we assume that *R* is nontrivial, both ker(R) and ker(R+id) are nonzero, and dim(ker(R))≥dim(ker(R+id)). Then, for n≅g, either g has a nontrivial Levi decomposition, or g is solvable.

*Case 1:* Assume that g has a nontrivial Levi decomposition, i.e., that $g≅sl_{2}(C)⋉r$. We claim that $sl_{2}(C)$ is a direct summand of g, i.e., $g≅sl_{2}(C)⊕r$, and that r is not isomorphic to $r_{3}(C)$. Then we can argue as follows. Because of Remark 2.12 of [12], g cannot be unimodular, except for g≅n. Thus r cannot be unimodular, so that g is isomorphic to $sl_{2}(C)⊕r_{3,\lambda }(C)$ with λ≠−1. On the other hand, all such algebras do arise by Proposition 2.6 and Proposition 4.7 of [11].

*Case 1a:* Suppose that $sl_{2}(C)$ is not contained in ker(R),ker(R+id) as a subalgebra. Then dim(ker(R+id))=1 and dim(ker(R))∈{1,2}. Let us assume, both have dimension 1. The other case goes similarly. Then we have $r=〈x_{1},x_{2},x_{3}〉,\ker(R)=〈x_{1}〉$ and $\ker(R+\text{id})=〈x_{2}〉$. Furthermore $\text{im}(R)≅sl_{2}(C)⋉〈x_{2},x_{3}〉$ and $\text{im}(R+\text{id})≅sl_{2}(C)⋉〈x_{1},x_{3}〉$ are five-dimensional subalgebras of n. By Table 3, $sl_{2}(C)$ is a direct summand of them. This implies that $sl_{2}(C)$ is also a direct summand in g. Since both ker(R) and ker(R+id) are ideals in r, we can exclude that r is isomorphic to $r_{3}(C)$, and we are done.

**Table 3.** Semisimple subalgebras and Levi decomposable subalgebras.

dim(h)	Representative	Isomorphism type
3	$〈X_{1},Y_{1},H_{1}〉,\;〈X_{1}+X_{2},Y_{1}+Y_{2},H_{1}+H_{2}〉$	$sl_{2}(C)$
4	$〈X_{1},Y_{1},H_{1},H_{2}〉,\;〈X_{1},Y_{1},H_{1},X_{2}〉$	$sl_{2}(C)⊕C$
5	$〈X_{1},Y_{1},H_{1},X_{2},H_{2}〉$	$sl_{2}(C)⊕r_{2}(C)$

*Case 1b:*$sl_{2}(C)$ is contained in one of ker(R),ker(R+id). Without loss of generality we may assume that $sl_{2}(C)\subseteq \ker(R)$. If $\ker(R)=sl_{2}(C)$, then $sl_{2}(C)$ is an ideal of g, and we have $g≅sl_{2}(C)⊕r$, where r≅im(R)≤n is not isomorphic to $r_{3}(C)$ by Table 2, and we are done. Thus we may assume that dim(ker(R))≥4. If *R* splits with subalgebras ker(R) and ker(R+id), then g≅ker(R)⊕ker(R+id), and dim(ker(R))+dim(ker(R+id))=6. By Table 3, $sl_{2}(C)$ is a direct summand of ker(R), and hence of g. So we have again $g≅sl_{2}(C)⊕r$, and r is not isomorphic to $r_{3}(C)$. If *R* is not split, it remains to consider the case dim(ker(R))=4 and dim(ker(R+id))=1. We have r=〈x,y,z〉 with $\ker(R)=sl_{2}(C)⊕〈x〉,\ker(R+\text{id})=〈y〉$ and $[y,sl_{2}(C)]=0$. Assume that $[z,sl_{2}(C)]\neq 0$. Then $sl_{2}(C)$ is not a direct summand of the five-dimensional subalgebra im(R+id) of n, which is a contradiction to Table 3. Thus we have $g≅sl_{2}(C)⊕r$. Since r has two disjoint one-dimensional ideals 〈x〉 and 〈y〉, it is not isomorphic to $r_{3}(C)$.

*Case 2:* Assume that g is solvable. Then im(R) and im(R+id) are solvable subalgebras of n of dimension at most 4 by Table 2. So we have dim(ker(R))≥dim(ker(R+id))≥2. Thus we have the following four cases:

$$\begin{array}{c} (2a)\;\dim(\ker(R))=4,\dim(\ker(R+\text{id}))=2,\\ (2b)\;\dim(\ker(R))=3,\dim(\ker(R+\text{id}))=3,\\ (2c)\;\dim(\ker(R))=3,\dim(\ker(R+\text{id}))=2,\\ (2d)\;\dim(\ker(R))=2,\dim(\ker(R+\text{id}))=2. \end{array}$$

For the cases (2a) and (2b), *R* is split since the dimensions add up to 6. Then g is a direct sum of two solvable subalgebras, which are both isomorphic to subalgebras of n. So we have $n=\ker(R)\dot{+}\ker(R+\text{id})$ and g=ker(R)⊕ker(R+id).

*Case 2a:* Since we have only $r_{2}(C)⊕r_{2}(C)$ as four-dimensional solvable subalgebra of n, we have $g≅r_{2}(C)⊕r_{2}(C)⊕C^{2}$, which is of type (3) for (λ,μ)=(0,0), or $g≅r_{2}(C)⊕r_{2}(C)⊕r_{2}(C)$, which is of type (4). Both cases can arise. For the first one we will show this in case (2b). For the second, it follows from Proposition 2.7 with $n=〈X_{1},H_{1},X_{2},H_{2}〉\dot{+}〈Y_{1},Y_{2}+H_{1}〉$.

*Case 2b:* We have $g≅r_{3,\lambda }(C)⊕r_{3,\mu }(C)$. The case (λ,μ)=(−1,−1) cannot arise by Theorem 3.3 of [11]. The cases (λ,μ)=(−1,μ) for μ≠−1 arise by Proposition 2.7 with

$$n=〈X_{1},X_{2},H_{1}-H_{2}〉\dot{+}〈Y_{1},Y_{2},H_{1}+\mu H_{2}〉.$$

The other cases with λ,μ≠−1 arise by Proposition 2.6 and Proposition 4.7 of [11].

*Case 2c:* Here g is isomorphic to $(r_{3,\lambda }(C)⊕r_{2}(C))⋊C$ or $(r_{3,\lambda }(C)⊕C^{2})⋊C$. In the first case, $r_{2}(C)⋊C≅\text{im}(R)$ is a solvable subalgebra of n, hence isomorphic to $r_{3,\nu }(C)$ by Table 2. So C acts trivially on $r_{2}(C)$, and $\text{im}(R+\text{id})≅r_{3,\lambda }(C)⋊C≅r_{2}(C)⊕r_{2}(C)$. Then $g≅r_{2}(C)⊕r_{2}(C)⊕r_{2}(C)$, which we have already considered in Case (2a). For $(r_{3,\lambda }(C)⊕C^{2})⋊C$ we need to distinguish λ=0 and λ≠0.

*Case 2c,*λ=0*:* By Proposition 2.3 we may assume that $\text{im}(R+\text{id})=〈X_{1},H_{1},X_{2},H_{2}〉$. Since ker(R) is an ideal of im(R+id) isomorphic to $r_{2}(C)⊕C$, we have $\ker(R)=〈X_{1},H_{1},X_{2}〉$. Let us consider the characteristic polynomial *χ_R_* of the linear operator *R* acting on n. By assumption on the kernels, $\chi_{R}(t)=t^{3}{\left(t+1\right)}^{2}(t-\rho )$.

*Case 2c,*λ=0,ρ≠0,−1*:* Then $R(x_{6})=\rho x_{6}$ for $x_{6}=H_{2}+\alpha H_{1}+\beta X_{1}+\gamma X_{2}$. Since ker(R+id) is an abelian two-dimensional subalgebra of n, we have

$$\ker(R+\text{id})=〈Y_{1}+\nu_{1}X_{1}+\nu_{2}H_{1},Y_{2}+\nu_{3}X_{2}+\nu_{4}H_{2}〉.$$

We want to compute [x,y] for *x* = *x*_6_ and y∈ker(R+id). By Proposition 2.13 we have, using $R(x_{6})=\rho x_{6}$

$$\begin{array}{l} \left[x,y]=\{R(x),y\}-\{R(y),x\}+\{x,y\right\}\\ =\{R(x),y\}\\ =\rho \{x,y\}. \end{array}$$

For $x_{6}=H_{2}+\alpha H_{1}+\beta X_{1}+\gamma X_{2}$ and y∈ker(R+id) this yields, using the Lie brackets of n in the standard basis $\left\{X_{1},Y_{1},H_{1},X_{2},Y_{2},H_{2}\right\}$,

(10) $$[x_{6},Y_{1}+\nu_{1}X_{1}+\nu_{2}H_{1}]=\rho ((2\alpha \nu_{1}-2\beta \nu_{2})X_{1}-2\alpha Y_{1}+\beta H_{1}),$$

(11) $$[x_{6},Y_{2}+\nu_{3}X_{2}+\nu_{4}H_{2}]=\rho ((2\nu_{3}-2\gamma \nu_{4})X_{2}-2Y_{2}+\gamma H_{2}).$$

Since ker(R+id) is an ideal in g and ρ≠0, both vectors lie again in ker(R+id). Comparing coefficients for the basis vectors we obtain

$$\beta =-2\alpha \nu_{2},\;\alpha (\nu_{1}+\nu_{2}^{2})=0,\;\gamma =-2\nu_{4},\;\nu_{3}=-\nu_{4}^{2}.$$

Suppose that α=0. Then $x_{6}=H_{2}-2\nu_{4}X_{2}$ and $〈X_{1},H_{1}〉≅r_{2}(C)$ is a direct summand of g. Therefore $g≅r_{2}(C)⊕C⊕r_{3,\mu }(C)$ with $C=〈Y_{1}+\nu_{1}X_{1}+\nu_{2}H_{1}〉$, $r_{3,\mu }(C)=〈X_{2},H_{2}-2\nu_{4}X_{2},Y_{2}+\nu_{4}H_{2}-\nu_{4}^{2}X_{2}〉,\mu =-(\rho +1)/\rho$, which we have already considered above. Hence we may assume that α≠0 and $\nu_{1}=-\nu_{2}^{2}$. Consider a new basis for g (note that we redefine *x*_6_) given by

$$\begin{array}{c} (x_{1},\ldots ,x_{6})=(X_{1},-\frac{1}{2}H_{1}+\nu_{2}X_{1},X_{2},Y_{1}+\nu_{2}H_{1}-\nu_{2}^{2}X_{1},Y_{2}+\nu_{4}H_{2}-\nu_{4}^{2}X_{2},\\ \frac{1}{2\rho }(H_{2}+\alpha H_{1}-2\alpha \nu_{2}X_{1}-2\nu_{4}X_{2})), \end{array}$$

with Lie brackets

$$[x_{1},x_{2}]=x_{1},\;[x_{1},x_{6}]=-\frac{\rho +1}{\rho }\alpha x_{1},\;[x_{3},x_{6}]=-\frac{\rho +1}{\rho }x_{3},\;[x_{4},x_{6}]=\alpha x_{4},\;[x_{5},x_{6}]=x_{6}.$$

This algebra is of type (5), if we replace *x*_6_ by $x_{6}+\frac{\alpha (\rho +1)}{\rho }x_{2}$. It arises for the triangular-split RB-operator *R* with $A_{-}=\ker(R)=〈x_{1},x_{2},x_{3}〉,\ker(R+\text{id})=〈x_{4},x_{5}〉$ and $A_{0}=〈x_{6}〉$, where $x_{6}=H_{2}-2\nu_{4}X_{2}$, with the action $R(x_{6})=\rho x_{6}$.

*Case 2c,*λ=0,ρ=−1*:* We may assume that there exists $x_{6}=Y_{2}+v$ such that $\left(R+\text{id})(x_{6})=\mu (H_{2}+\alpha H_{1}+\beta X_{1}+\gamma X_{2}\right)$ for some nonzero *μ* and some α,β,γ∈C. Since ker(R+id) is an abelian subalgebra we obtain α=β=0 and $\ker(R+\text{id})=〈H_{2}+\gamma X_{2},Y_{1}+\nu_{1}X_{1}+\nu_{2}H_{1}〉$. Then we may choose $x_{6}=Y_{2}+\kappa X_{2}+\nu_{3}H_{1}+\nu_{4}X_{1}$. Then

$$\begin{array}{l} \left[x_{6},H_{2}+\gamma X_{2}]=\{R(x_{6}),H_{2}+\gamma X_{2}\right\}\\ =\{(R+\text{id})(x_{6})-x_{6},H_{2}+\gamma X_{2}\}\\ =-\{Y_{2}+\kappa X_{2},H_{2}+\gamma X_{2}\}\\ =-2Y_{2}+2\kappa X_{2}+\gamma H_{2}. \end{array}$$

This is not contained in ker(R+id), which is a contradiction to the fact that ker(R+id) is an ideal.

*Case 2c,*λ=0*, ρ = 0:* Then we have $R(H_{2})=\alpha H_{1}+\beta X_{1}+\gamma X_{2}\neq 0$ and $\ker(R+\text{id})=〈Y_{1}+\nu_{1}X_{1}+\nu_{2}H_{1},Y_{2}+\nu_{3}X_{2}+\nu_{4}H_{2}〉$. Since $\left[H_{2},Y_{2}+\nu_{1}X_{1}+\nu_{2}H_{1}]=\{\gamma X_{2},Y_{2}+\nu_{1}X_{2}+\nu_{2}H_{2}\right\}$ is in ker(R+id), we obtain γ=0. Since $\left[H_{2},Y_{1}+\nu_{1}X_{1}+\nu_{2}H_{2}]=\{\alpha H_{1}+\beta X_{1},Y_{1}+\nu_{1}X_{1}+\nu_{2}H_{1}\right\}$ is in ker(R+id), we obtain $\alpha (\nu_{1}+\nu_{2}^{2})=0$ and $\beta =-2\alpha \nu_{2}$. Since $R(H_{2})\neq 0$ we have $\alpha \neq 0,\nu_{1}=-\nu_{2}^{2}$ and $R(H_{2})=\alpha H_{1}-2\alpha \nu_{2}X_{1}$. Consider a new basis for g given by

$$(x_{1},\ldots ,x_{6})=(X_{1},-\frac{1}{2}H_{1}+\nu_{2}X_{1},X_{2},Y_{1}+\nu_{2}H_{1}-\nu_{2}^{2}X_{1},Y_{2}+\nu_{3}X_{2}+\nu_{4}H_{2},-\frac{1}{2}H_{2}),$$

with Lie brackets

$$[x_{1},x_{2}]=x_{1},\;[x_{1},x_{6}]=\alpha x_{1},\;[x_{3},x_{6}]=x_{3},\;[x_{4},x_{6}]=-\alpha x_{4}.$$

This algebra is of type (3), if we replace *x*_6_ by $x_{6}-\alpha x_{2}$.

*Case 2c,*λ≠0*:* Then we have $\ker(R)=〈X_{1},X_{2},-\frac{1}{2}(H_{1}+\lambda H_{2})〉$. We again have $\chi_{R}(t)=t^{3}{\left(t+1\right)}^{2}(t-\rho )$, where we distinguish the cases ρ≠0,−1,ρ=−1 and *ρ* = 0.

*Case 2c,*λ≠0,ρ≠0,−1*:* Then we may assume that $R(x_{6})=\rho x_{6}$ for $x_{6}=H_{2}+\alpha H_{1}+\beta X_{1}+\gamma X_{2}$. As ker(R+id) is abelian, we have $\ker(R+\text{id})=〈Y_{1}+\nu_{1}X_{1}+\nu_{2}H_{1},Y_{2}+\nu_{3}X_{2}+\nu_{4}H_{2}〉$. Since $V=\ker(R)⊕\ker(R+\text{id})⊕〈x_{6}〉$, the two elements $H_{1}+\lambda H_{2}$ and $H_{2}+\alpha H_{1}$ need to be linearly independent, i.e., 1−αλ≠0. By (10) and (11) we obtain $\gamma =-2\nu_{4},\beta =-2\alpha \nu_{2},\nu_{3}=-\nu_{4}^{2}$, and $\alpha (\nu_{1}+\nu_{2}^{2})$. Suppose that α=0. Then $x_{6}=H_{2}-2\nu_{4}X_{2}$. Consider a new basis for g given by

$$(x_{1},\ldots ,x_{6})=(Y_{1}+\nu_{1}X_{1}+\nu_{2}H_{1},X_{1},X_{2},-\frac{1}{2}(H_{1}+\lambda H_{2}),Y_{2}-\nu_{4}^{2}X_{2}+\nu_{4}H_{2},-\frac{1}{2(\rho +1)}H_{2}),$$

with Lie brackets

$$[x_{2},x_{4}]=x_{2},\;[x_{3},x_{4}]=\lambda x_{3},\;[x_{3},x_{6}]=x_{3},\;[x_{4},x_{6}]=-\lambda \nu_{4}x_{3},\;[x_{5},x_{6}]=-\frac{\rho }{1+\rho }x_{5}.$$

This is an algebra of type (6), if we replace *x*_4_ by $x_{4}+\lambda \nu_{4}x_{3}$.

Now we assume that α≠0. Consider a new basis for g given by

$$\begin{array}{c} (x_{1},\ldots ,x_{6})=(X_{1},X_{2},-\frac{1}{2}(H_{1}+\lambda H_{2}),Y_{2}-\nu_{4}^{2}X_{2}+\nu_{4}H_{2},Y_{1}-\nu_{2}^{2}X_{1}+\nu_{2}H_{1},\\ -\frac{1}{2(\rho +1)}(H_{2}-2\nu_{4}X_{2}+\alpha (H_{1}-2\nu_{2}X_{1}))), \end{array}$$

with Lie brackets

$$\begin{array}{c} {}[x_{1},x_{3}]=x_{1},\;[x_{2},x_{3}]=\lambda x_{2},\;[x_{1},x_{6}]=\alpha x_{1},\;[x_{2},x_{6}]=x_{2},\\ {}[x_{3},x_{6}]=-\alpha \nu_{2}x_{1}-\lambda \nu_{4}x_{2},\;[x_{4},x_{6}]=\delta x_{4},\;[x_{5},x_{6}]=\alpha \delta x_{5}, \end{array}$$

where $\delta =-\frac{\rho }{\rho +1}$. Replacing *x*_6_ by $\frac{1}{\delta }(x_{6}-\alpha \nu_{2}x_{1}-\nu_{4}x_{2}-\alpha x_{3})$ we obtain the Lie brackets

$$[x_{1},x_{3}]=x_{1},\;[x_{2},x_{3}]=\lambda x_{2},\;[x_{2},x_{6}]=\alpha \prime x_{2},\;[x_{4},x_{6}]=x_{4},\;[x_{5},x_{6}]=\alpha x_{5},$$

where

$$\alpha \prime =\frac{1-\alpha \lambda }{\delta }=\frac{\left(\rho +1)(\alpha \lambda -1\right)}{\rho }.$$

Note that α′≠0 and α′≠αλ−1 by assumption. In other words, $\alpha \neq \frac{\alpha \prime +1}{\lambda }$. Consider a new basis for g given by

$$(x_{1}^{\prime },\ldots ,x_{6}^{\prime })=(x_{2},x_{1},\frac{1}{\lambda }x_{3},x_{4},x_{5},x_{6}-\frac{\alpha \prime }{\lambda }x_{3}),$$

with Lie brackets

$$[x_{1}^{\prime },x_{3}^{\prime }]=x_{1}^{\prime },\;[x_{2}^{\prime },x_{3}^{\prime }]=\lambda \prime x_{2}^{\prime },\;[x_{2}^{\prime },x_{6}^{\prime }]=-\alpha \prime \lambda \prime x_{2}^{\prime },\;[x_{4}^{\prime },x_{6}^{\prime }]=x_{4}^{\prime },\;[x_{5}^{\prime },x_{6}^{\prime }]=\alpha x_{5}^{\prime },$$

where $\lambda \prime =\frac{1}{\lambda }$. This is of type (7). Since $r_{3,\lambda }(C)≅r_{3,\lambda \prime }(C)$, one may check that we do not only have $\alpha \neq \frac{\alpha \prime +1}{\lambda }$, but also α≠λ−α′. For $\frac{\alpha \prime +1}{\lambda }\neq \lambda -\alpha \prime$ we obtain no restriction for α. However, for $\frac{\alpha \prime +1}{\lambda }=\lambda -\alpha \prime$ we obtain λ=−1 or λ=α′+1, which excludes both (λ,α′,α)=(−1,α′,−α′−1) and (λ,α′,α)=(λ,λ−1,1). Rewriting this in the parameters of the Lie brackets from type (7), we obtain all cases except for (λ,α′,α)=(λ,λ−1,1) with λ≠−1. These PA-structures arise by a triangular-split RB-operator with $A_{-}=\ker(R),A_{+}=\ker(R+\text{id})$ and $A_{0}=〈x_{6}〉$ with the action $R(x_{6})=\rho x_{6},\rho \neq 0,-1$.

*Case 2c,*λ≠0,ρ=−1*:* This leads to a contradiction in the same way as case 2*c* with λ=0,ρ=−1.

*Case 2c,*λ≠0*,ρ = 0:* We have $R(H_{2})=\alpha X_{1}+\beta X_{2}+\gamma (H_{1}+\lambda H_{2})$ and $\ker(R+\text{id})=〈x_{4},x_{5}〉$ with $x_{4}=Y_{1}+\nu_{1}X_{1}+\nu_{2}H_{1},x_{5}=Y_{2}+\nu_{3}X_{2}+\nu_{4}H_{2}$. Similarly to (10),(11) we obtain $R(H_{2})=\gamma (H_{1}-2\nu_{2}X_{1})+\gamma \lambda (H_{2}-2\nu_{4}X_{2})$. This implies that γ≠0 and $x_{4}=Y_{1}-\nu_{2}^{2}X_{1}+\nu_{2}H_{1},x_{5}=Y_{2}-\nu_{4}^{2}X_{2}+\nu_{4}H_{2}$. By setting *x*_1_ = *X*_1_, *x*_2_ = *X*_2_, $x_{3}=-\frac{1}{2}(H_{1}+\lambda H_{2})$ and $x_{6}=\frac{1}{2\gamma }H_{2}$ we obtain a new basis for g with Lie brackets

$$\begin{array}{c} {}[x_{1},x_{3}]=x_{1},\;[x_{2},x_{3}]=\lambda x_{2},\;[x_{1},x_{6}]=-x_{1},\;[x_{2},x_{6}]=\delta x_{2},\\ {}[x_{3},x_{6}]=\nu_{2}x_{1}+\lambda^{2}\nu_{4}x_{2},\;[x_{4},x_{6}]=x_{4},\;[x_{5},x_{6}]=\lambda x_{5}, \end{array}$$

where $\delta =-\frac{1+\lambda \gamma }{\gamma }$ with δ≠−λ. Replacing *x*_6_ by $x_{6}+\nu_{2}x_{1}+\lambda \nu_{4}x_{2}+x_{3}$ we obtain the brackets

$$[x_{1},x_{3}]=x_{1},\;[x_{2},x_{3}]=\lambda x_{2},\;[x_{2},x_{6}]=\alpha_{1}x_{2},\;[x_{4},x_{6}]=x_{4},\;[x_{5},x_{6}]=\lambda x_{5}$$

with $\alpha_{1}=\delta +\lambda =-\frac{1}{\gamma }$. This is of type (7) with $\alpha_{2}=\lambda$. It arises by the triangular-split RB-operator with $A_{-}=〈x_{1},x_{2}〉,A_{+}=〈x_{4},x_{5}〉$ and $A_{0}=〈u,v〉$, with $u=\frac{1}{\gamma }(H_{2}-2\nu_{4}X_{2})$ and $v=H_{1}-2\nu_{2}X_{1}+\lambda (H_{2}-2\nu_{4}X_{2})$, and the action *R*(*u*) = *v*, *R*(*v*) = 0.

*Case 2d:* Suppose that one of the kernels ker(R) and ker(R+id) is nonabelian. Without loss of generality, let us assume that $\ker(R)≅r_{2}(C)$. Write g≅(ker(R)⊕ker(R+id))⋉〈a,b〉. Then ker(R)⋉〈a〉 is a three-dimensional solvable subalgebra of im(R+id). By Table 2, we see that it is isomorphic to $r_{2}(C)⊕C$. In this case there exist nonzero a′∈ker(R)⊕〈a〉 and b′∈ker(R)⊕〈b〉 such that [a′,ker(R)]=[b′,ker(R)]=0. Then g≅ker(R)⊕(ker(R+id)⊕〈a′,b′〉) with $\ker(R)≅r_{2}(C)$, and $\ker(R+\text{id})⊕〈a\prime ,b\prime 〉≅r_{2}(C)⊕r_{2}(C)$ by Table 2. Hence we obtain $g≅r_{2}(C)⊕r_{2}(C)⊕r_{2}(C)$, which is of type (4).

So we may assume that $\ker(R)≅\ker(R+\text{id})≅C^{2}$. Then the characteristic polynomial of *R* has the form $\chi_{R}(t)=t^{2}{\left(t+1\right)}^{2}(t-\rho_{1})(t-\rho_{2})$.

*Case 2d,*$\rho_{1},\rho_{2}\neq 0,-1$: Suppose first that either $\rho_{1}\neq \rho_{2}$ or that $\rho_{1}=\rho_{2}$ and the eigenspace is two-dimensional. Then by Proposition $3.4,n=\ker(R)\dot{+}\ker(R+\text{id})\dot{+}〈x_{5}^{\prime },x_{6}^{\prime }〉$ with linearly independent eigenvectors $x_{5}^{\prime },x_{6}^{\prime }$ corresponding to the eigenvalues *ρ*_1_ and *ρ*_2_. Since ker(R) is an abelian ideal in $\text{im}(R+\text{id})=〈X_{1},H_{1},X_{2},H_{2}〉$, we may assume that $\ker(R)=〈X_{1},X_{2}〉$ and $[x_{5}^{\prime },x_{6}^{\prime }]=0$. The decomposition $n=\ker(R+\text{id})\dot{+}\text{im}(R+\text{id})$ shows that ker(R+id) has a basis $x_{3}=Y_{1}+\alpha H_{1}+\nu_{3}X_{1}$, $x_{4}=Y_{2}+\beta H_{2}+\nu_{4}X_{2}$. Since $[x_{5}^{\prime },x_{6}^{\prime }]=0$, we have $x_{5}^{\prime }=H_{1}+\nu_{1}X_{1}+\xi_{1}(H_{2}+\nu_{2}X_{2})$, $x_{6}^{\prime }=H_{2}+\nu_{2}X_{2}+\xi_{2}(H_{1}+\nu_{1}X_{1})$ with $\xi_{1}\xi_{2}\neq 1$. So we have by (10) and (11) $x_{3}=Y_{1}-\frac{\nu_{1}}{2}H_{1}-\frac{\nu_{1}^{2}}{4}X_{1}$, $x_{4}=Y_{2}-\frac{\nu_{2}}{2}H_{2}-\frac{\nu_{2}^{2}}{4}X_{2}$. Consider a basis for g given by

$$(x_{1},\ldots ,x_{6})=(X_{1},X_{2},x_{3},x_{4},-\frac{1}{2(1+\rho_{1})}x_{5}^{\prime },-\frac{1}{2(1+\rho_{2})}x_{6}^{\prime }),$$

with Lie brackets

$$\begin{array}{c} {}[x_{1},x_{5}]=x_{1},\;[x_{1},x_{6}]=\xi_{2}x_{1},\;[x_{2},x_{5}]=\xi_{1}x_{2},\;[x_{2},x_{6}]=x_{2},\\ {}[x_{3},x_{5}]=\gamma x_{3},\;[x_{3},x_{6}]=\delta \xi_{2}x_{3},\;[x_{4},x_{5}]=\gamma \xi_{1}x_{4},\;[x_{4},x_{6}]=\delta x_{4}, \end{array}$$

where $\gamma =-\frac{\rho_{1}}{\rho_{1}+1}$, $\delta =-\frac{\rho_{2}}{\rho_{2}+1}$ with γ,δ≠0,−1 and $\xi_{1}\xi_{2}\neq 1$. This is type (8a). It arises by the triangular-split RB-operator *R* with $A_{-}=〈X_{1},X_{2}〉,A_{+}=〈x_{3},x_{4}〉$ and $A_{0}=〈x_{5},x_{6}〉$, where *R* acts on *A*_0_ by $R(x_{5})=\rho_{1}x_{5}$ and $R(x_{6})=\rho_{2}x_{6}$. Note that for $\nu_{2}=\xi_{2}=0$ and $\xi_{1}\neq 0$ we get type (7) without the restriction $\left(\lambda ,\alpha_{1},\alpha_{2})\neq (\lambda ,\lambda -1,1\right)$ for λ≠−1, which we had in Case 2c, λ≠0,ρ≠0,−1.

Suppose now that $\rho_{2}=\rho_{1}\neq 0,-1$, and the eigenspace for *ρ*_1_ is one-dimensional. Let $R(x_{5}^{\prime })=\rho_{1}x_{5}^{\prime }$ and $R(x_{6}^{\prime })=x_{5}^{\prime }+\rho_{1}x_{6}^{\prime }$. In the same way as before we have $x_{5}^{\prime }=H_{1}+\nu_{1}X_{1}+\xi (H_{2}+\nu_{2}X_{2})$, $x_{6}^{\prime }=\kappa (H_{2}+\nu_{2}X_{2})$ with κ≠0 and $x_{3}=Y_{1}-\frac{\nu_{1}}{2}H_{1}-\frac{\nu_{1}^{2}}{4}X_{1}$, $x_{4}=Y_{2}-\frac{\nu_{2}}{2}H_{2}-\frac{\nu_{2}^{2}}{4}X_{2}$. Consider a basis for g given by

$$(x_{1},\ldots ,x_{6})=(X_{1},X_{2},x_{3},x_{4},-\frac{1}{2(1+\rho_{1})}x_{5}^{\prime },-\frac{1}{2(1+\rho_{1})}x_{6}^{\prime }),$$

with Lie brackets

$$\begin{array}{c} {}[x_{1},x_{5}]=x_{1},\;[x_{1},x_{6}]=(\gamma +1)x_{1},\;[x_{2},x_{5}]=\xi x_{2},\;[x_{2},x_{6}]=(\kappa +\xi +\gamma \xi )x_{2},\\ {}[x_{3},x_{5}]=\gamma x_{3},\;[x_{3},x_{6}]=-(\gamma +1)x_{3},\;[x_{4},x_{5}]=\gamma \xi x_{4},\;[x_{4},x_{6}]=(\kappa \gamma -\xi -\gamma \xi )x_{4}, \end{array}$$

where $\gamma =-\frac{\rho_{1}}{\rho_{1}+1}\neq 0,-1$ and κ≠0. This is type (8b). It arises by the triangular-split RB-operator *R* with $A_{-}=〈X_{1},X_{2}〉,A_{+}=〈x_{3},x_{4}〉$ and $A_{0}=〈x_{5},x_{6}〉$, where *R* acts on *A*_0_ by $R(x_{5})=\rho_{1}x_{5}$ and $R(x_{6})=x_{5}+\rho_{1}x_{6}$.

*Case 2d,*$\rho_{1}=\rho_{2}=0$*:* We have $g=\ker(R+\text{id})\dot{+}\text{im}(R+\text{id})$ and we can assume that $\ker(R)=〈X_{1},X_{2}〉$ and $\ker(R+\text{id})=〈Y_{1}+\nu_{1}X_{1}+\nu_{2}H_{1},Y_{2}+\nu_{3}X_{2}+\nu_{4}H_{2}〉$. Suppose first that $R(v)=X_{1}$ and $R(w)=X_{2}$ for some *v*, *w*. Then

$$[Y_{1}+\nu_{1}X_{1}+\nu_{2}H_{1},v]=\{Y_{1}+\nu_{1}X_{1}+\nu_{2}H_{1},X_{1}\}=-H_{1}+2\nu_{2}X_{1}\in \ker(R+\text{id}),$$

which is a contradiction. Otherwise we see from the possible Jordan forms of *R* that there exist *v*, *w* with $R(v)=\alpha X_{1}+\beta X_{2}\neq 0$ and *R*(*w*) = *v*. This leads to a contradiction in the same way.

*Case 2d,*$\rho_{1}=0,\rho_{2}\neq 0,-1$*:* This case is analogous to the second part of the case before.

*Case 2d,*$\rho_{1}=0,\rho_{2}=-1$*:* As above we may assume that $\text{im}(R+\text{id})=〈X_{1},X_{2},H_{1},H_{2}〉$ and $\ker(R)=〈X_{1},X_{2}〉$, and $\alpha H_{1}+\beta H_{2}+\gamma X_{1}+\delta X_{2}\in \ker(R+\text{id})\cap \text{im}(R+\text{id})$ for some α,β,γ,δ∈C. Since ker(R+id) is abelian, we may assume that $\ker(R+\text{id})=〈H_{1}+\nu_{1}X_{1},Y_{2}+\nu_{2}X_{2}+\nu_{3}H_{2}〉$ for some $\nu_{1},\nu_{2},\nu_{3}\in C$. Let $v\in \ker(R^{2})$ such that $R(v)=\nu_{4}X_{1}+\nu_{5}X_{2}\neq 0$. Then

$$\left[v,Y_{2}+\nu_{2}X_{2}+\nu_{3}H_{2}]=\{\nu_{4}X_{1}+\nu_{5}X_{2},Y_{2}+\nu_{2}X_{2}+\nu_{3}H_{2}\}=\nu_{5}(H_{2}-2\nu_{3}X_{2})\in \ker(R+\text{id}\right)$$

implies that $\nu_{5}=0$. By $\left[v,H_{1}+\nu_{1}X_{1}]=\{\nu_{4}X_{1},H_{1}+\nu_{1}X_{1}\}=-2\nu_{4}X_{1}\in \ker(R+\text{id}\right)$ we obtain $\nu_{4}=0$, which is a contradiction to R(v)≠0.□

Remark 4.2. The algebras from different types are nonisomorphic, except for algebras of type (8), which have intersections with types (3) and (7) for certain parameter choices.
